# Classification of Cancer Primary Sites Using Machine Learning and Somatic Mutations

**DOI:** 10.1155/2015/491502

**Published:** 2015-10-11

**Authors:** Yukun Chen, Jingchun Sun, Liang-Chin Huang, Hua Xu, Zhongming Zhao

**Affiliations:** ^1^Department of Biomedical Informatics, Vanderbilt University School of Medicine, Nashville, TN 37203, USA; ^2^School of Biomedical Informatics, The University of Texas Health Science Center at Houston, Houston, TX 77030, USA; ^3^Department of Psychiatry, Vanderbilt University School of Medicine, Nashville, TN 37212, USA; ^4^Department of Cancer Biology, Vanderbilt University School of Medicine, Nashville, TN 37232, USA

## Abstract

An accurate classification of human cancer, including its primary site, is important for better understanding of cancer and effective therapeutic strategies development. The available big data of somatic mutations provides us a great opportunity to investigate cancer classification using machine learning. Here, we explored the patterns of 1,760,846 somatic mutations identified from 230,255 cancer patients along with gene function information using support vector machine. Specifically, we performed a multiclass classification experiment over the 17 tumor sites using the gene symbol, somatic mutation, chromosome, and gene functional pathway as predictors for 6,751 subjects. The performance of the baseline using only gene features is 0.57 in accuracy. It was improved to 0.62 when adding the information of mutation and chromosome. Among the predictable primary tumor sites, the prediction of five primary sites (large intestine, liver, skin, pancreas, and lung) could achieve the performance with more than 0.70 in *F*-measure. The model of the large intestine ranked the first with 0.87 in *F*-measure. The results demonstrate that the somatic mutation information is useful for prediction of primary tumor sites with machine learning modeling. To our knowledge, this study is the first investigation of the primary sites classification using machine learning and somatic mutation data.

## 1. Introduction

Cancer is a complex disease, which is driven by the combination of genetic, environmental, and lifestyle factors. Among these factors, the combination of multiple genes driving cancer development varies considerably among cancer types and patients [1]. During the past decade, investigation of mutations at both large-scale and specific loci has been made in order to increase our knowledge of the molecular heterogeneity in this complex disease. Notably, several large-scale, network-based cancer genome projects have generated multidimensional and genome-wide data. These projects include The Cancer Genome Atlas (TCGA) [2], Wellcome Trust Sanger Institute's Cancer Genome Project [3], and the International Cancer Genome Consortium (ICGC) [4]. These projects have dramatically advanced cancer research, especially in its genetics and genomics [5]. A cancer somatic mutation landscape, primarily focusing on nucleotide change patterns (e.g., C->T) and mutation signatures in the cancer genomes, has been released to the community [6]. Among these achievements, some have been translated into molecular diagnosis, better prognosis, and new targeted therapies. For example, the germline mutations in* BRCA1* and* BRCA2* confer high risks to breast and ovarian cancers [7]. Their genotyping is used to determine susceptibility to breast and ovarian cancer [8–10]. To monitor the treatment, the increased expression level of circulating tumor marker, human epidermal growth factor receptor 2 (HER2), is used to determine the treatment of a monoclonal antibody trastuzumab in breast cancer [11–13]. However, cancer is strongly heterogeneous, and the cancer classification is a critical first step in the further investigation of the pathology of cancer and the development of effective treatments.

For cancer classification, the fundamental method is mainly based on the cell of origin or their histological types [14]. During the last two decades, molecular profiling has been unveiled for classification of cancer types and subtypes, as well as assessment of heterogeneity of cancer samples [15]. For example, in breast cancer, recent studies that are mainly based on microarray-based gene expression data and unbiased hierarchical clustering have identified several molecular subtypes: basal-like, ErbB2^+^, normal breast-like, luminal subtype A, and luminal subtype B [16, 17]. Further gene expression profiling was found to be effective on identifying even more specific subtypes in triple negative breast cancer type [18]. As massive amount of genomic, transcriptomic, and proteomic data in cancer cells and patients becomes available, an integrated model of cancer classification was recently proposed to capture the known attributes of cancer by integrating morphology, cancer stem cells, proteomics, and genomics [19]. However, as other data integration schemes, it presents a big challenge to develop an effective and comprehensive method for cancer classification.

Recently, next-generation sequencing approaches have been applied to cancer studies, including whole genome sequencing, whole exome sequencing, targeted gene sequencing, whole transcriptome sequencing, genome-wide microRNA sequencing, and epigenomics, providing the highest resolution (base-pair resolution) of genetic and genomic information in cancer. These datasets provide us an unprecedented opportunity on systematic and integrated investigation of molecular mechanisms of cancer. For example, Vogelstein et al. systematically analyzed the mutation landscapes in 96 cancer types reported from 127 publications, providing deep insights into the cancer genomic architecture [20]. Among these datasets, somatic mutation data in cancer genomes has been accumulated dramatically, which makes it possible to discover novel cancer genes and mutations [21–23], draw mutational landscapes among multiple cancers [6, 24], and explore the molecular mechanisms of tumorigenesis [25]. In this study, we hypothesized that features from the massive amount of somatic mutations could act as effective contributors for cancer site classification. Moreover, another goal of the study is to search for the associations between cancer sites and mutation features in a larger scale using machine learning.

In this study, we proposed a novel cancer site classification framework by investigating somatic mutations through machine learning approaches. The somatic mutation information includes (1) patient information, (2) mutation-associated genes, and (3) mutation-associated chromosomes. We extracted these types of information from the database COSMIC (Catalogue of Somatic Mutations In Cancer) [26]. We further integrated the mutation-associated gene function using gene pathways from the database KEGG (Kyoto Encyclopedia of Genes and Genomes) [27]. Our evaluation showed that the combination of the somatic mutation, mutation-associated gene, and mutation-associated chromosome features achieved the best performance of cancer site classification.

## 2. Methods and Materials

### 2.1. Overview of Study Design

**Figure 1:**
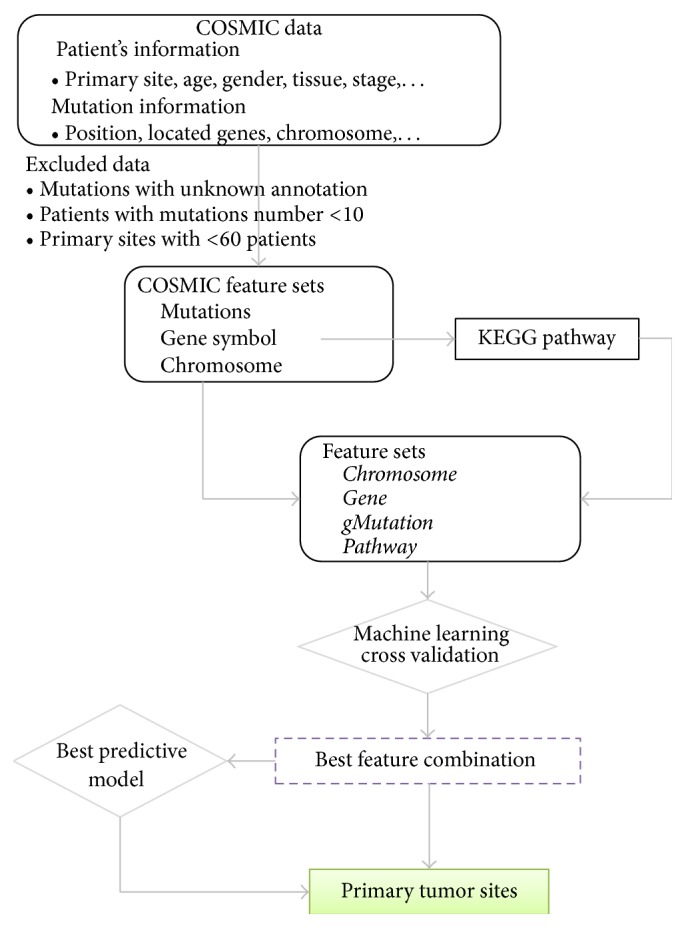
Study design using somatic mutations to classify primary tumor sites by machine learning model. In order to precisely represent the mutations, we generated a feature* gMutation* by binding mutations with their corresponding gene symbols.

The main purpose of this study is to test if the somatic mutation features and mutation-related information are useful or have the power to predict the primary cancer site since more than a million somatic mutations in cancer genomes have been reported, collected, and systematically analyzed. To address this important question, we took advantage of the data in COSMIC, which is the most comprehensive, annotation-based database for the somatic mutations from numerous patients with cancer type information.  Figure 1 illustrates the study design.

### 2.2. Data Sources

The COSMIC database is established to collect, store, and display somatic mutations and related information extracted from the primary literature on human cancers as well as those identified from cancer genome projects [26]. The COSMIC data provides a consistent view of histology and tissue ontology with the mutation information. We downloaded the data from COSMIC website on April 18, 2014. The downloaded data contained 990,529 samples, 25,660 genes, 1,292,597 coding mutations, 1,528,225 noncoding variations, and 11,330 references.

To normalize the gene names to the gene official symbols, we took a two-step strategy. First, we utilized the mutation positions from COSMIC data to map the gene regions using the UCSC Genome Browser based on the GRCh37 genome annotation [28]. Thus, we obtained three sets of gene names: (1) gene names without position information in COSMIC; (2) gene names with position information in COSMIC but could not be matched to the UCSC Genome Browser; and (3) gene names with the matched information (gene names and locations) in the UCSC Genome Browser. Finally, we utilized the Entrez Gene Table to match these gene names to their corresponding official gene symbols [29].

To clean the data, we removed the records that do not have the information about gene name, sample ID, primary site, or mutation description. Additionally, we removed the mutations that were involved in fusion genes because they do not have a single-mutation position. Eventually, the filtered dataset contained 230,255 patients, 22,111 unique genes, and 1,760,846 mutations.

KEGG pathway database manually collects and annotates the molecular interactions and regulations among genes and then draws pathway maps [27]. We downloaded the data on May 21, 2014, from website (http://www.kegg.jp/kegg/). We extracted the genes from their involved pathways. In total, there are 285 human pathways and 6,503 genes involved in 22,573 pathway-gene relationships. Then, we matched the mutation-associated genes into the pathways.

### 2.3. Datasets and Features

In this study, we mainly explored the somatic mutations and their relative information for cancer primary site classification. From the filtered data obtained above, we extracted 7,251 patients who had at least ten mutations. Patients with a very small number of mutations would be more likely outliers in the dataset and fail to provide sufficient information for a model to distinguish the final label with other patients. These limitations increase the difficulty in training a good predictive model. On the other hand, patients with a larger number of mutations more likely have common features and thus induce better training to find a more reliable pattern in the model. We chose ten as the threshold because the filtered patients set of over seven thousand is large enough for machine learning experiments and the number of features generated for each patient based on the threshold of ten does not discourage the modeling process.

We further filtered out several minority classes of primary tumor sites. Each of them has less than 60 patients in the dataset, such as “Bone,” “Meninges,” and “Eye.” Thus, the final set of 6,751 patients was chosen to be used in this study. These patients were diagnosed to be one type of cancer among the 17 primary tumor sites.  Table 1 shows the distribution of the patients with the primary tumor sites.

From the COSMIC data, we collected mutations and their corresponding mutated genes and chromosomes to represent the genetic characteristics of each patient. As a result, our process led to twelve unique types into four categories (e.g., substitution, insertion, deletion, and complex) and eight more specific descriptions (e.g., substitution-nonsense, substitution-missense, substitution-coding silent, substitution-intronic, insertion-in frame, insertion-frameshift, deletion-frameshift, and deletion-frameshift) according to the mutation description in the COSMIC and our filtering procedure.  Table 2 includes their detailed descriptions. In our dataset, these mutations could be mapped to 21,286 unique genes in all patients.

Instead of directly using individual mutation description, we bound them with their corresponding gene symbols to precisely represent the mutations. It resulted in 79,865 unique combos of gene symbols and mutation descriptions in the dataset, such as “CHDC2_Insertion-Frameshift,” “SPEN_Complex,” and “SP1_Substitution-Missense.” In this paper, we use “*gMutation*” to represent the feature set of mutations associated with genes. In our study design, we considered* gene* symbol and* gMutation* as two different features.* Gene* symbol feature represents a larger range of biological activity at the gene level while the* gMutation* feature represents a more precise feature at the mutation level located in a specific gene region. Despite the fact that both features are not independent, they could represent cancer patients at two different levels. Thus, we utilized them together in the prediction modeling.

Since the human somatic mutation landscape is related to chromosome [30], we further considered the* Chromosome* as the third feature in our study. The human genome includes 22 autosomes (1–22), two sex chromosomes (X, Y), and one mitochondrial genome (MT). Thus, there are a total of 25 features included in the* Chromosome* feature set.

Besides the mutation-related information, we further integrated the KEGG dataset to provide the functional knowledge of the genes involved in the patients' mutations. There are 285 unique pathways for the 21,286 genes.

Therefore, in this study, we defined four features:* Gene*,* gMutation*,* Chromosome*, and* Pathway*. Furthermore, we attempted to find the optimal combination of these four feature sets for the best prediction performance using the* Gene* feature as the baseline.

### 2.4. Machine Learning Experiments

In the data we collected, each sample contains an array of features that are present in one patient. We present all the collected features in all patients as a feature vector in the machine learning fashion. All features in the vector were represented by binary values; namely, “1” represents present while “0” represents not present. Then, we constructed a data matrix, in which each row includes all the features for one patient while each column includes one type of feature for all patients.

With respect to the classification method, we implemented a one-versus-all multiclass classification schema to identify the primary tumor site based on patients' mutation-associated features and the gene pathway feature. For each primary tumor site, we trained a binary classifier that could distinguish the class belonging to the site versus the one that does not. Each classifier was a support vector machine (SVM) with linear kernel implemented by LIBLINEAR [31]. Given the 17 trained binary classifiers, we predicted the primary tumor site for an undiagnosed patient to be a class from the corresponding classifier with the highest confidence value, which is the distance to the hyperplane from the trained SVM. For the experimental parameter set in LIBLINEAR, we used “L1-regularized L2-loss support vector classification” as the solver for the multiclass classification task. L1-regularization was selected because the gene mutation based feature set is large (>100,000 features among <7,000 samples) and sparse (very few nonzero entries in the data matrix).

We performed the multiclass classification experiments on the* Gene* feature (baseline) and six different combinations of four feature sets in the fashion of 10-fold cross validation (see Section 2.5). To avoid being overoptimistic on the modeling, we did not optimize the parameter set of the linear SVM model. That is, the parameter set (L1-regularization, L2-loss function, and cost = 10, among others) was fixed through the entire 10-fold cross validation experiments. We chose the combination of feature sets with the best performance in accuracy for the generation of our best predictive model. Then, we applied the best model to predict the primary tumor site over 17 cancer candidates. The performance over each primary tumor site was evaluated by precision, recall, and *F*-measure.

### 2.5. Evaluation

We conducted experiments by 10-fold cross validation. All patient samples were split into ten folds with stratification so that the class distribution in each is much similar to the one from the original dataset. We alternately treated one fold as the test set and the other as the training set. Then we did the predictive model training and testing 10 times. Eventually, each patient would have a diagnosis of the primary tumor site by the predictive model. We computed the accuracy as global metric to evaluate different feature combinations. We also evaluated the performance of prediction on each primary tumor site by precision, recall, and *F*-measure:

(1) $$\begin{array}{c} \text{Accuracy}=\frac{\sum_{y_{i}}^{}\text{TP}\left(y_{i}\right)}{\sum_{y_{i}}^{}\text{Pred}\left(y_{i}\right)},\\ \text{Precision}\left(y_{i}\right)=\frac{\text{TP}\left(y_{i}\right)}{\text{Pred}\left(y_{i}\right)},\\ \text{Recall}\left(y_{i}\right)=\frac{\text{TP}\left(y_{i}\right)}{\text{True}\left(y_{i}\right)},\\ F\text{-measure}\left(y_{i}\right)=\frac{2*\text{Precision}\left(y_{i}\right)*\text{Recall}\left(y_{i}\right)}{\text{Precision}\left(y_{i}\right)+\text{Recall}\left(y_{i}\right)}, \end{array}$$

where *y*_*i*_ is one of the given primary tumor sites or classes; TP(*y*_*i*_) is the number of true positives of the given class *y*_*i*_ predicted by the model; Pred(*y*_*i*_) is the number of the predictions of the given class; True(*y*_*i*_) is the number of true positives of the given class in the dataset.

We also used microaverage and macroaverage methods to report the accuracy. In the microaverage accuracy (miAccuracy), TP(*y*_*i*_) in the numerator is the summation of true positives of a given class over tenfold and the denominator is equivalent to the number of total patients. In the macroaverage accuracy (maAccuracy), we generated the accuracy for each and reported the mean and standard deviation (SD) over 10-fold results.

## 3. Results

Following the study design in Figure 1, we first rigorously filtered the data at the mutation, patient, and tumor site levels to reduce the data noises and improve the predictive performance. Thus, among the 990,529 samples in the downloaded data, we only recruited 6,751 patients in our study. To test if the higher-level functional knowledge is useful to improve the performance, we integrated the gene pathway information into the feature set. Then, we identified the best feature combination by cross validation. Finally, based on the best feature combination, we developed one best predictive model set and applied it to predict the primary tumor sites.

### 3.1. Identification of the Best Feature Combination

We have trained seven predictive models using different combinations of feature sets. The specific features for each combination, sizes of features, and the accuracies as their global scores are shown in Table 3. We considered the predictive model using* Gene* feature only as our baseline, which achieved 0.57 in accuracy. With one additional feature set (*gMutation*,* Chromosome*, or* Pathway*), the model achieved slightly better (0.58, 0.58, and 0.60, resp.). If we combined three types of feature sets, the model reached the best performance (0.62) when features* Gene*,* gMutation*, and* Chromosome* were combined. However, when we added* Pathway* as the fourth feature set, the accuracy dropped back to 0.60. We also tested other combinations, but none of them had better achievement than the best model (data not shown).

### 3.2. Prediction of Primary Tumor Site

Using the best model set, we predicted the whole dataset using 10-fold cross validation and evaluated the performance on every primary tumor site by precision, recall, and *F*-measure.  Table 4 shows the performance of the best predictive model set using the combination of three features (*Gene* +* gMutation* +* Chromosome*) over each tumor site.

The average precision and recall were 0.70 and 0.49, respectively. This predictive model could achieve the precision of 0.75 or higher in 8 out of 17 primary tumor sites, recall of 0.60 or higher for 8 out of 17, and *F*-measure of 0.60 or higher for 9 out of 17.

## 4. Discussion 

In this study, we performed a systematic exploration of the somatic mutations and their related features for cancer classification using a machine learning approach and the most comprehensive somatic mutation dataset so far. The study filtered the somatic mutation data from COSMIC, identified the best feature combination, and predicted the primary tumor sites using the machine learning methods.

Machine learning approaches have been applied to cancer prognosis and prediction [32]. In our study, the performance of primary tumor site prediction is strongly correlated with its sample size (correlation coefficient = 0.58). Therefore, increasing the sample size could be a major way to improve the performance. However, for some specific sites, this is not always true. For example, the primary tumor site “skin” only contains 2.74% samples in the dataset and ranked the 13th over the 17 primary sites studied based on the sample percentage in this study, but its model ranked 3rd in *F*-measure (0.73). The primary site “Lung” has the largest percentage of samples, but it was ranked 5th in *F*-measures. To discover the underlying reason for this observation, we further computed the coverage rate of the genes that occurred in the true positives identified by the predictive model for each primary tumor site. The coverage rate of a gene X in a primary tumor site is the ratio between the counts of the true positives where the gene X occurred and the total number of true positives. The top four primary tumor sites in prediction (large intestine, liver, skin, and pancreas) share the pattern of “Top Heavy” in coverage rate distribution (with max coverage rate over 50%), while “Lung” has distribution over genes closer to uniform (max coverage rate of 16%). Therefore, without as many as relatively strong associated genes, it is harder to predict “Lung” than these top four primary sites, although “Lung” has the most number of training samples.

For the bottom four primary sites with the smallest sample size, the performance by the model tended to be poorest. Specifically, “Oesophagus,” “Urinary tract,” “Upper aerodigestive tract,” and “Stomach” had smallest numbers of samples, and they were also ranked at the bottom according to *F*-measure values. For those primary tumor sites with a large number of samples but without excellent prediction performance (e.g., “Lung,” “Breast,” “Haematopoietic and lymphoid tissue”), they had a much better recall (all 0.75) than others, but poor precision (0.66, 0.50, and 0.50, resp.).

One important output of this study is the best feature combination (*Gene* +* gMutation* +* Chromosome*) compared to other combinations. Though the three features were directly related to mutation feature, they reflected three features at differently genetic architecture at three levels, namely, DNA-sequence, DNA function, and DNA organization. This observation indicated that, with more detailed information on mutation, the best combination could contribute the cancer class classification. The result illustrated that the somatic mutation could be used to predict primary tumor sites in the individual way or the integrative way.

To test if the high-level function-associated features could improve the performance of cancer site classification, we explored the KEGG pathway that mutation-associated genes are involved in. However, in our study, there is no improvement of performance by integrating the* Pathway* feature into other features. One possible reason is that a gene can be involved in multiple pathways; this is especially true for cancer genes, which have important function and regulation in biological system and often involve in multiple signaling pathways. If only one pathway had a high association with one cancer type, the additional pathways could lead to the noise for the prediction of such cancer type. Moreover, the* Pathway* feature increased the dimensions of our feature space rather than refined our predictors. Finally, pathway size varies greatly, but this characteristic was not taken into account in the pathway analysis in this study. We would use a better way to represent the KEGG pathway feature set. Instead of using binary (zero or one) representation, for example, we could use quantitative value between zero and one to represent the involvement of the mutated genes in the pathway so that the pathways with higher number of mutated genes involved would have higher weights as predictors.

Our prediction model utilized the* Gene* feature as the baseline.  Table 5 summarizes the genes that have been used in the model. The number of genes used in the modeling varied greatly, which might be one reason that the performance for multiple primary sites is much different.

**Figure 2:**
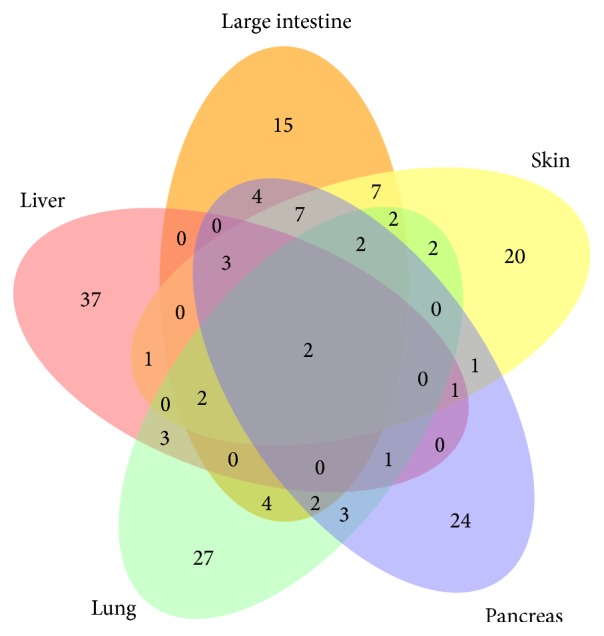
Comparison among five sets of the top 50 genes used in the machine learning modeling for five primary tumor sites (large intestine, liver, lung, pancreas, and skin).

Among the 17 primary tumor sites, five primary tumor sites achieved better performance, according to their *F*-measure values (>0.70). They are “large intestine,” “liver,” “skin,” “pancreas,” and “lung.” To illustrate the common and specific genes in these five tumor sites, we selected the top 50 genes according to the counts of genes that occurred in the true positive patients identified by the model for each primary tumor site.  Figure 2 shows the overlap among the five sets of the genes in five primary sites. The number of common genes among the five primary sites is different, which might reflect their histological relationship among them. For example, the “large intestine” has 25 common genes to “skin,” 20 common genes to “pancreas,” 14 common genes to “lung,” and 7 common genes to “liver.” Notably, there are only 2 (*TTN* and* LRP1B*) common genes among the five sets of the genes. Searching the COSMIC (version 69) dataset, the gene* TTN* has 3,403 mutations in the unique 1,881 samples. However, only 17 mutations have been reported in more than three samples. This gene is the longest human gene, and its cancer risk remains unclear [33, 34]. The gene* LRP1B*, which encodes one of the low density lipoproteins (LDL), is reported as a novel candidate tumor suppressor gene [35]. It has 1,302 mutations in the unique 939 samples. Only two mutations have been reported in more than three samples. Besides the common genes, each primary site has its own mutation-associated genes. It will be useful further to check them for further understanding of their genetic architectures.

In this exploratory study, we demonstrated that the somatic mutation information could be used for cancer classification. As the first attempt for prediction of cancer sites, we have seen many opportunities to improve the performance based on the genetic and genomic information in future work. First, refinement of the features might improve the performance of machine learning experiments in several ways. (1) The first is identification and analysis of the most frequently mutated genes across multiple primary sites. (2) The second is reducing redundancy of feature sets by automatic dimension reduction techniques. We can use two types of methods. One is the algorithms without the label information, such as, principle component analysis [36], latent Dirichlet allocation [37], and sufficient dimension reduction [38]. Another is the algorithm using the label information including HITON [39] and random forest. (3) The third is normalization of* Gene* feature by the gene length. For example, the gene* TTN* is the longest known coding gene and thus might accrue mutations by chance that happened in many tumor sites. (4) The fourth is integrating more molecular features (e.g., methylation, gene expression, and gene direct interacting relationship). And (5) considering specific functional mutations or mutational features may improve prediction power. For example, mutations of specific nucleotide change (e.g., C->T in melanoma), or mutations causing critical amino acid changes or protein structure alterations, will likely be more informative. Second, adding information from normal subjects in our modeling might boost the performance. Adding the normal subjects as a control group to the modeling process is more applicable on a clinical perspective. Finally, applying multiple machine learning algorithms to our task might be robust for evaluation of prediction performance. Many other machine learning methods for cancer classification have been reported. Dettling [40] combined bagging and boosting to precisely classify cancerous malignancies at an early stage using microarray data. Liu et al. [41] also utilized microarray data and machine learning for cancer classification research. Their proposed classifier Recursive Feature Addition with a gene selection method Lagging Prediction Peephole Optimization outperformed popular learning machines such as SVM, Naïve Bayes classifier, and random forest. Hofree et al. [42] introduced a network-based stratification (NBS) algorithm to stratify cancer into informative subtypes by grouping patients together with mutations in similar network regions. They have demonstrated that the identified subtypes are predictive of many clinical outcomes such as patient survival, response to therapy, or tumor histology. We are interested in using the networks with subtype labels generated by NBS to identify the primary tumor site. We plan to design and evaluate better machine learning methods and explore deep learning techniques [43] for better cancer classification with the resource of big data.

## 5. Conclusion

In conclusion, our application of the machine learning technique to somatic mutations could predict some primary tumor sites, such as the large intestine, liver, skin, pancreas, and lung. Since treatment of cancer does rely on not only the known cancer site, but also the underlying molecular profiles (e.g., cancer driver mutations) and cancer cells migrate to multiple sites at metastasis stage, the prediction of cancer sites based on mutation profiles may be helpful for the enhancement of molecular therapeutics development. This study represents the first large-scale prediction of primary tumor site using comprehensive, publicly available somatic mutations through a machine learning approach.

## Figures and Tables

**Table 1:** Distribution of primary tumor sites.

Primary tumor site	Number of patients	Percentage (%)
Lung	970	14.43
Breast	967	14.39
Large intestine	654	9.73
Haematopoietic and lymphoid tissue	644	9.58
Kidney	491	7.31
Ovary	490	7.29
Liver	400	5.95
Central nervous system	377	5.61
Prostate	374	5.56
Endometrium	261	3.88
Pancreas	252	3.75
Autonomic ganglia	222	3.30
Skin	184	2.74
Oesophagus	174	2.59
Urinary tract	110	1.64
Upper aerodigestive tract	91	1.35
Stomach	60	0.89

**Table 2:** Mutation description.

Mutation description	Definition
Substitution	A mutation involving the substitution of a single nucleotide
Substitution-nonsense	A substitution mutation resulting in a termination codon, foreshortening the translated peptide
Substitution-missense	A substitution mutation resulting in an alternate codon, altering the amino acid at this position only
Substitution-coding silent	A synonymous substitution mutation which encodes the same amino acid as the wild type codon
Substitution-intronic	A substitution mutation outside the coding domains; no interpretation is made as to its effect on splice sites or nearby regulatory regions
Insertion	An insertion of novel sequence into the gene
Insertion-in frame	An insertion of nucleotides which does not affect the gene's translation frame, leaving the downstream peptide sequence intact
Insertion-frameshift	An insertion of novel sequence which alters the translation frame, changing the downstream peptide sequence (often resulting in premature termination)
Deletion	A deletion of a portion of the gene's sequence
Deletion-in frame	A deletion of nucleotides which does not affect the gene's translation frame, leaving the downstream peptide sequence intact
Deletion-frameshift	A deletion of nucleotides which alters the translation frame, changing the downstream peptide sequence (often resulting in premature termination)
Complex	A compound mutation which may involve multiple insertions, deletions, and substitutions

**Table 3:** Micro- and macroaveraged accuracies of seven combinations of gene symbols with three other features.

Feature combination	Number of features	miAccuracy	maAccuracy (mean)	maAccuracy (SD)
*Gene* (baseline)	21,286	0.57	0.57	0.019
*Gene + gMutation *	101,151	0.58	0.58	0.019
*Gene + Pathway *	21,571	0.58	0.58	0.010
*Gene + Chromosome *	21,311	0.60	0.60	0.022
*Gene + gMutation + Pathway *	101,436	0.60	0.60	0.013
*Gene + gMutation + Chromosome *	101,176	0.62	0.62	0.021
*Gene + gMutation + Chromosome + Pathway *	101,461	0.60	0.60	0.015

Note: miAccuracy represents the microaverage accuracy; maAccuracy represents the macroaverage accuracy, which is reported in mean and standard deviation (SD) over 10 accuracies from 10-fold cross validation.

**Table 4:** Precision, recall, and *F*-measure for the best predictive model using “*Gene*,” “*gMutation*,” and “*Chromosome*” on each primary tumor site.

Primary tumor site	Precision	Recall	*F*-measure
Large intestine	0.88	0.85	0.87
Liver	0.88	0.72	0.79
Skin	0.91	0.61	0.73
Pancreas	0.75	0.67	0.71
Lung	0.66	0.75	0.70
Endometrium	0.91	0.52	0.67
Kidney	0.72	0.62	0.66
Haematopoietic and lymphoid tissue	0.50	0.75	0.60
Breast	0.50	0.75	0.60
Central nervous system	0.63	0.51	0.56
Ovary	0.40	0.49	0.44
Prostate	0.46	0.35	0.40
Autonomic ganglia	0.45	0.28	0.34
Oesophagus	0.81	0.20	0.31
Urinary tract	0.83	0.09	0.16
Upper aerodigestive tract	1.00	0.05	0.10
Stomach	0.60	0.05	0.09

**Table 5:** Summary of genes and samples used in the primary tumor site prediction.

Primary tumor site	Number of genes	Number of true positives
Large intestine	18,066	555
Liver	19,778	287
Skin	10,898	113
Pancreas	3,364	170
Lung	18,423	724
Endometrium	18,234	137
Kidney	10,601	302
Haematopoietic and lymphoid tissue	14,545	723
Breast	6,327	486
Central nervous system	2,773	192
Ovary	8,169	238
Prostate	5,875	132
Autonomic ganglia	1,425	62
Oesophagus	6,200	34
Urinary tract	3,288	10
Upper aerodigestive tract	1,013	5
Stomach	86	3
